# Mortalin/glucose-regulated protein 75 promotes the cisplatin-resistance of gastric cancer via regulating anti-oxidation/apoptosis and metabolic reprogramming

**DOI:** 10.1038/s41420-021-00517-w

**Published:** 2021-06-11

**Authors:** Yi Dai, Fan Li, Yuwen Jiao, Guoguang Wang, Tian Zhan, Yunwei Xia, Hanyang Liu, Haojun Yang, Jianping Zhang, Liming Tang

**Affiliations:** 1 Department of General Surgery, The Second Affiliated Hospital, Nanjing Medical University, Nanjing, China; 2 Department of General Surgery, The Affiliated Changzhou No. 2 Hospital of Nanjing Medical University, Changzhou, China

**Keywords:** Gastric cancer, Tumour biomarkers

## Abstract

Platinum drug treatment is one of the most predominant chemotherapeutic strategies for patients with gastric cancer (GC). However, the therapeutic effect is less than satisfactory, largely due to the acquired resistance to platinum drugs. Therefore, a better understanding of the underlying mechanisms can greatly improve the therapeutic efficacy of GC. In this study, we aimed to investigate the chemo-resistance related functions/mechanisms and clinical significance of glucose-regulated protein 75 (GRP75) in GC. Here, our data showed that compared with SGC7901 cells, the expression of GRP75 was markedly higher in cisplatin-resistance cells (SGC7901^CR^). Knockdown of GRP75 abolished the maintenance of mitochondrial membrane potential (MMP) and inhibited the nuclear factor erythroid-2-related factor 2 (NRF2), phosphatidylinositol 3 kinase/protein kinase B (PI3K/AKT), hypoxia-inducible factor 1α (HIF-1α), and c-myc, which resulted in blocking the activation of their downstream targets. These processes attenuated the anti-oxidation/apoptosis abilities and altered the metabolic reprogramming in SGC7901^CR^ cells, leading to re-sensitizing these cells to cisplatin. However, overexpression of GRP75 in SGC7901 cells caused the opposite effects. A xenografts model confirmed the abovementioned results. In GC patients receiving platinum chemotherapy and a meta-analysis, a high level of GRP75 was positively associated with aggressive characteristics and poor prognosis including but not limited to gastrointestinal cancers, and was an independent predictor for overall survival. Collectively, our study indicated that GRP75 was involved in the cisplatin-resistance of GC and that GRP75 could be a potential therapeutic target for restoring the drug response in platinum-resistance cells and a useful additive prognostic tool in guiding clinical management of GC patients.

## Introduction

Gastric cancer (GC) was the first leading incidence and second leading mortality of digestive system cancers in China^1^. Current treatments for advanced GC were the surgery operation combined with systemic chemotherapy, but the long-term survival rate was less than satisfactory because of the high post-surgical recurrence^2^. In clinical practice, platinum drugs were one of the first-line agents for advanced GC chemotherapy^3^. However, acquired resistance to drugs always occurred after multiple cycles of platinum-based treatment and indicates a poor prognosis. Therefore, illuminating the potential mechanisms, and identifying the novel therapeutic strategies to overcome platinum drugs-resistance in GC patients were urgently essential.

Glucose-regulated protein 75 (GRP75) was the stress-inducible molecular chaperones that belong to the heat shock protein family^4^. Overexpression of GRP75 was closely associated with tumor progression in various human cancers^5–7^. Here, via exploring a meta-analysis, we found that a high level of GRP75 indicated a significantly poor prognosis in several digestive system cancers (colorectal cancer, cholangiocarcinoma, and pancreatic cancer). For drug resistance, inhibition of GRP75 reversed the cisplatin and doxorubicin resistance in hepatocellular carcinoma and ovarian cancer^8,9^. GRP75 classically sequestrated the p53 in the cytoplasm, leading to the inactivation of p53 function and suppressing the apoptosis^10^. A previous study reported that GRP75 positive tumors had a worse prognosis compared with GRP75 negative tumors in GC with normal p53 function^11^. However, p53 was one of the most frequently mutated genes in GC (affecting more than 50% of patients)^12–15^. So we hypothesized that in addition to the classical repression of p53 activity, GRP75 might be involved in inducing/maintaining the platinum drugs-resistance in GC via employing a p53 independent manner.

In this study, the higher expression of GRP75 contributed to cisplatin-resistance in SGC7901 cells and in a xenografts model. Mechanistically, GRP75 induced/maintained cisplatin-resistance via regulating the anti-oxidation/apoptotic abilities and metabolic reprogramming properties. In GC patients, overexpression of GRP75 contributed to the aggressive characteristics and poor prognosis. Our results indicated that GRP75 promoted the cisplatin-resistance in GC and could be a biomarker for predicting the response to platinum drug treatment. Targeting GRP75 might provide a new understanding of GC systemic chemotherapy.

## Results

### Effects of GRP75 on cisplatin-resistance in GC

**Fig. 1:**
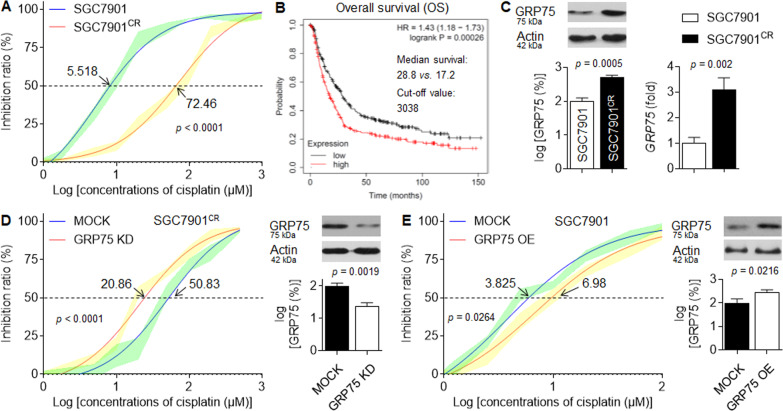
Effects of GRP75 on cisplatin-resistance in GC. **A** SGC7901 or SGC7901^CR^ cells were treated by different concentrations of cisplatin (0 to 10^3^ μM) for 24 h, respectively. The cell viabilities were determined in triplicate, and the IC_50_s were calculated. **B** Prognostic significance of GRP75 [Affy ID: 200691_s_at (HSPA9), http://kmplot.com/analysis/index.php?p=service&cancer=gastric]. **C** Western blot analysis and qPCR analysis in triplicate of the levels of GRP75 protein (left) and mRNA (right). **D** and **E** SGC7901^CR^ cells were transfected by scrambled or GRP75 siRNA, while SGC7901 cells were transfected by scrambled or GRP75 plasmids. Cells were treated with different concentrations of cisplatin for 24 h. The cell viabilities were determined in triplicate, and the IC50s were calculated. Western blot analysis of the GRP75 in GC cells.

Cell viability assays were employed to verify the resistance of SGC7901^CR^ cells, the IC_50_s (μM) of cisplatin for SGC7901 and SGC7901^CR^ cells were: 5.518 vs. 72.46 (Fig. 1A). Based on KM-Plotter databases, the increased expression of GRP75 indicated a poor prognosis (Fig. 1B). Moreover, markedly increased GRP75 expressions in SGC7901^CR^ cells compared with its parental SGC7901 cells (Fig. 1C), suggesting that GRP75 might contribute to the cisplatin-resistance. To verify this hypothesis, SGC7901^CR^ cells were transfected by scramble- or GRP75 siRNA, the IC50s (μM) of cisplatin for scrambled- or GRP75 siRNA transfected SGC7901^CR^ cells were: 50.83 vs. 20.86, respectively (Fig. 1D). In contrast, overexpression of GRP75 by transfecting the GRP75 plasmids into SGC7901 cells showed that the IC50s of the cisplatin for scramble- or GRP75 plasmids transfected SGC7901 cells were 3.825 vs. 6.98 (Fig. 1E). Collectively, these results revealed that GRP75 played crucial roles in maintaining/inducing the cisplatin-resistance in GC, but the mechanisms remained further investigation.

### Potential mechanisms underlying GRP75 caused cisplatin-resistance

**Fig. 2:**
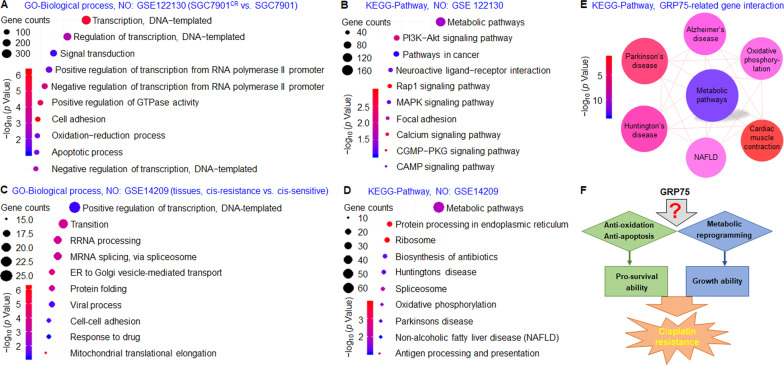
Potential mechanisms underlying GRP75 caused cisplatin-resistance. **A** and **B** GO-Biological process and KEGG-pathway analyses of DEGs from the GSE122130 dataset. **C** and **D** GO-Biological process and KEGG-pathway analysis of DEGs from the GSE14209 dataset. **E** Network-Analyst was used to analyze the PPI network of GRP75 and its interactors according to the KEGG database. **F** A hypothetic scheme showed that GRP75 might promote survival and growth of GC which leading to cisplatin-resistance via anti-oxidation/apoptosis and metabolic reprogramming, but the mechanism was unknown.

We downloaded microarray datasets GSE122130 (SGC7901^CR^ vs. SGC7901) and GSE14209 (tissues, cisplatin-resistant vs. cisplatin-sensitive) from GEO, then we performed GO-Biological process and KEGG-Pathway enrichment analysis based on DAVID, and the top 10 results were shown in Fig. 2A–D. The essential differences of biological processes between SGC7901^CR^ cells and SGC7901 cells included oxidation-reduction, apoptotic process (Fig. 2A), KEGG-Pathway enrichment analysis found that DEGs sets were closely correlated with metabolic pathways and phosphatidylinositol 3 kinase/protein kinase B (PI3K/AKT) pathway (Fig. 2B). Response to the drug was included in the essential differences of biological processes between cisplatin-resistant and cisplatin-sensitive tumor tissues and the metabolic pathway was also the core link in KEGG-Pathway enrichment analysis (Fig. 2C, D). Besides, a network of 60 proteins that significantly interacted with GRP75 were constructed using the String database, then KEGG-Pathway analysis performed that metabolic pathways played an important role between GRP75 and its interactors (Fig. 2E). Based on these results, we surmised that anti-oxidation/apoptosis and metabolic reprogramming might promote survival and growth of GC which leading to cisplatin-resistance. However, the mechanisms of GRP75 participation in regulation needed further study (Fig. 2F).

### Effects of GRP75 on anti-oxidation and anti-apoptosis

**Fig. 3:**
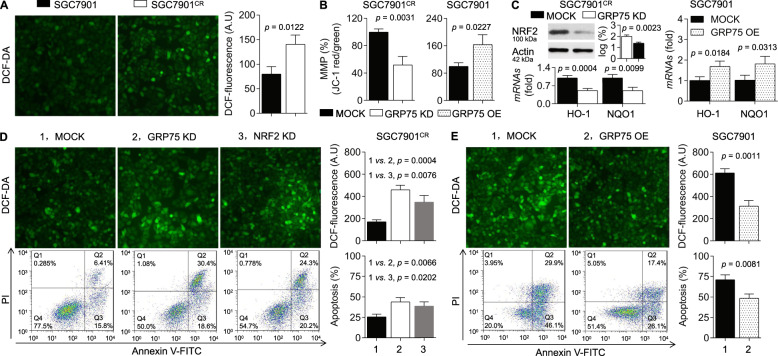
Effects of GRP75 on anti-oxidation and anti-apoptosis. **A** The intracellular ROS levels of SGC7901^CR^ or SGC7901 cells were determined in triplicate. **B**–**E** SGC7901^CR^ cells were transfected by scrambled or GRP75 siRNA, while SGC7901 cells were transfected by scrambled or GRP75 plasmids. After then, they were treated with 5 or 2.5 μM of cisplatin for 24 h. **B** MMP of GC cells was measured and calculated. **C** Western blot analysis of the levels of NRF2; and qPCR analysis in triplicate of the expressions of HO-1 and NQO-1. **D** and **E** The intracellular ROS levels and apoptosis rates (early apoptosis plus late apoptosis) were determined in triplicate.

Here, the intracellular ROS level was elevated in SGC7901^CR^ cells in comparison with its parental counterparts (Fig. 3A), suggesting that SGC7901^CR^ cells were exposed to relatively higher oxidative stress conditions. A previous study revealed that GRP75 was involved in the stabilization of MMP, an important source of ROS generation^16^. Here, knockdown of GRP75 abolished the maintenance of MMP in SGC7901^CR^ cells induced by cisplatin, and overexpression of GRP75 had the opposite effect in SGC7901 cells (Fig. 3B). Then, we validated the relationship between GRP75 and anti-oxidation and anti-apoptosis. As shown in Fig. 3C, knockdown of GRP75 decreased the level of nuclear factor erythroid-2-related factor 2 (NRF2) and its downstream target genes (HO-1 and NQO-1) in SGC7901^CR^ cells, and overexpression of GRP75 showed the opposite effect in SGC7901 cells. Moreover, knockdown of GRP75 or NRF2 in SGC7901^CR^ cells further enhanced the intracellular ROS generations, apoptosis (Fig. 3D), and caspase-3 activities (Supplementary Fig. S1) induced by cisplatin. In contrast, overexpression of GRP75 in SGC7901 cells significantly attenuated cisplatin-induced ROS generations, cell apoptosis (Fig. 3E), and caspase-3 activities (Supplementary Fig. S1). These results revealed that GRP75 maintained/induced cisplatin-resistance might be via inducing anti-oxidation and anti-apoptosis in GC cells.

### Effects of GRP75 on metabolic reprogramming

**Fig. 4:**
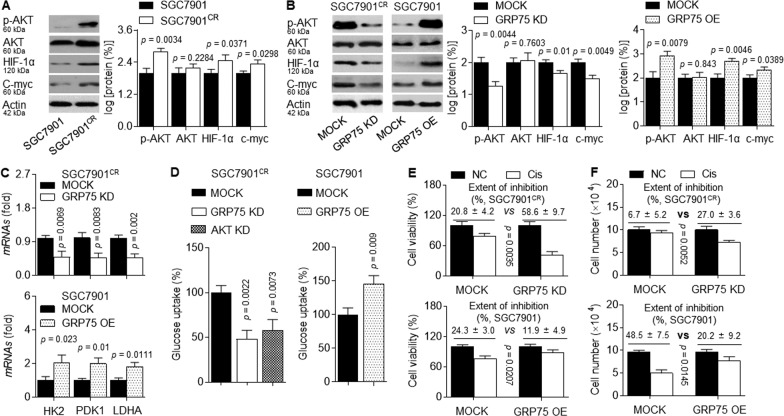
Effects of GRP75 on metabolic reprogramming. **A** Western blot (left) and quantitative analysis (right) of the levels of p-AKT, HIF-1α, and c-myc proteins in GC cells. **B**–**F** SGC7901^CR^ cells were transfected by scrambled or GRP75 siRNA, while SGC7901 cells were transfected by scrambled or GRP75 plasmids. After then, they were treated with 5 or 2.5 μM of cisplatin for 24 h. **B** Western blot (left) and quantitative analysis (right) of p-AKT, HIF-1α, and c-myc proteins. **C** qPCR analysis in triplicate of HK2, PDK1, and LDHA mRNAs. **D** Glucose uptake ability was evaluated and quantitatively calculated in triplicate. **E** and **F** The cell viabilities and cell numbers were determined, and the extent of inhibition was calculated in triplicate.

Next, we further investigated whereby GRP75 induced alteration of metabolic reprogramming. Increasing evidences showed that the metabolism of tumor cells was not an abnormal change in a single metabolic pathway, but a reprogramming of the entire cellular metabolic network^17^. Many classical oncogenes or signaling pathways were involved directly or indirectly in metabolic reprogramming, such as PI3K/AKT, hypoxia-inducible factor 1α (HIF-1α), c-myc and so on^9,18,19^. Therefore, we verified whether GRP75 was involved in the metabolic reprogramming of GC cells. As shown in Fig. 4A, we observed raised p-AKT, HIF-1α, and c-myc levels in SGC7901^CR^ cells compared with its parental SGC7901 cells. Moreover, in SGC7901^CR^ cells, knockdown of GRP75 decreased the cisplatin-induced p-AKT, HIF-1α, c-myc protein levels, and their downstream targets related to glycolysis (HK2: hexokinase 2; PDK1: pyruvate dehydrogenase kinase 1; and LDHA: lactate dehydrogenase A chain). On the contrary, overexpression of GRP75 in SGC7901 cells showed the opposite effect (Fig. 4B, C). Further, knockdown of GRP75 or AKT in SGC7901^CR^ cells showed a significant decrease in glucose uptake and cell viability/growth induced by cisplatin; however, overexpression of GRP75 in SGC7901 cells markedly increased the ability of glucose uptake and cell viability/growth induced by cisplatin (Fig. 4D–F). Collectively, these data indicated that GRP75 maintained/induced cisplatin-resistance in GC cells might be via participating in p-AKT, HIF-1α, and c-myc mediated metabolic reprogramming.

### Confirmation of the in vitro data in a xenograft model

**Fig. 5:**
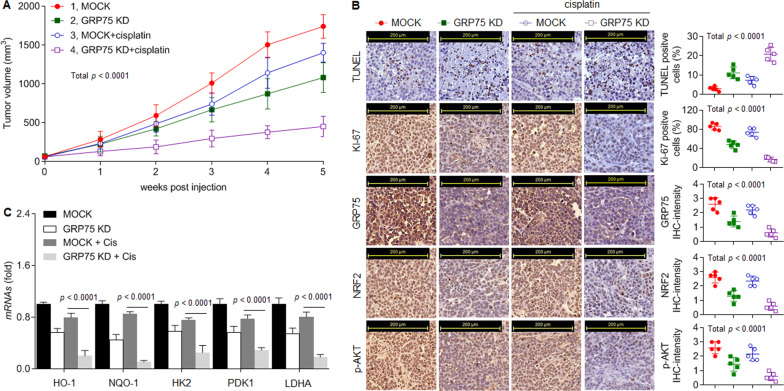
Confirmation of the in vitro data in a xenograft model. The SGC7901^CR^ cells xenograft tumors were treated by GRP75 siRNA alone, cisplatin alone, or cisplatin plus GRP75 siRNA. **A** The volumes of xenografts tumors in different treatments described above. **B** Tunnel and IHC staining (Note: each point represented the mean of one xenografts tumor section calculating in 5 high-power fields). **C** qPCR analysis in triplicate of the expressions of HO-1, NQO-1, HK2, PDK1, and LDHA mRNAs in xenografts tumors.

Then we investigated the potential clinical relevance of GRP75 in vivo. The xenograft data indicated that treatment with cisplatin alone or knockdown of GRP75 alone could inhibit the tumor growth; however, cisplatin treatment combining with GRP75 knockdown significantly facilitated the cisplatin-induced inhibition of tumor growth (Fig. 5A). Moreover, IHC and qPCR assays showed that cisplatin plus GRP75 siRNA significantly decreased the expressions of Ki67, GRP75, NRF2, p-AKT, and downstream targets compared with cisplatin treatment alone, but increased the apoptosis (as determined by TUNEL staining, Fig. 5B, C). Collectively, these results indicated that, via regulating the anti-oxidation/anti-apoptosis abilities and metabolic reprogramming, GRP75 stimulated the in vivo survival and growth which in turn leading to cisplatin-resistance of GC.

### Identification of GRP75 as a characteristic cancer-promoting factor and the clinical significance of GRP75 in GC

**Fig. 6:**
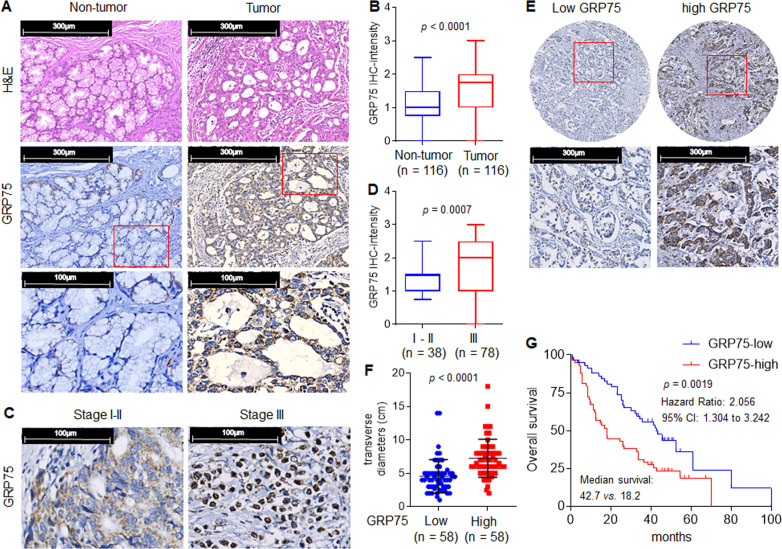
Identification of GRP75 as a characteristic cancer-promoting factor and the clinical significance of GRP75 in GC. **A** and **B** H&E, IHC staining, and IHC-intensity of GRP75 in tumors and adjacent tissues. **C** and **D** IHC staining and IHC-intensity of GRP75 in different TNM stages. **E** IHC staining of GRP75 in tumors with low and high levels. **F** The transverse diameters of tumors in GRP75 low and high groups. **G** Kaplan-Meier analysis of the prognostic significances of GRP75 in these samples.

We then evaluated GRP75 expression in consecutive sections of GC samples. As shown in Fig. 6A, compared with adjacent non-tumor gastric tissues, a considerable elevation of GRP75 expression was observed in GC tissues. Overexpression of GRP75 was demonstrated in GC tissues by IHC-intensity score (Fig. 6B). Stronger staining for GRP75 was also observed with increasing TNM Classification of malignant tumors stage (Fig. 6C, D). Then, we divided these 116 GC specimens into two groups (“GRP75 low” vs. “GRP75 high”, according to the IHC-intensity, Fig. 6E). The transverse diameters of tumors in the “GRP75 high” group were significantly larger than those in the “GRP75 low” group (Fig. 6F). We further validated the clinical prognosis of GRP75 in GC. Kaplan-Meier survival analysis also showed that GC patients in the “GRP75 high” group had a worse overall survival than those in the “GRP75 low” group (Fig. 6G). Multivariate analysis identified that GRP75 was an independent predictor for overall survival (Table 1). In summary, these results suggested that GRP75 had a characteristic role in leading GC progression, cisplatin-resistance, and poor prognosis.

**Table 1:** Univariate and multivariate analyses of factors associated with overall survival of GC patients (*n* = 116).

Clinical variables	Overall survival	
	HR (95% Cl)	*p-*value
Univariate analysis		
Age (≤60 vs. >60)	1.471 (0.883 to 2.449)	0.183
Gender (male vs. female)	1.019 (0.569 to 1.829)	0.950
Tumor size (≤5 cm vs. >5 cm)	1.997 (1.193 to 3.344)	0.009
Differentiation (well vs. poor)	2.002 (1.151 to 3.482)	0.014
Vascular invasion (negative vs. positive)	1.458 (0.822 to 2.584)	0.197
pTNM stage (I/II vs. III)	3.248 (1.594 to 6.617)	<0.0001
GRP75 (low vs. high)	2.817 (1.646 to 4.821)	<0.0001
Multivariate analysis		
pTNM stage (I/II vs. III)	2.221 (1.016 to 4.855)	0.045
GRP75 (low vs. high)	2.075 (1.151 to 3.740)	0.015

All patients were received platinum-based chemotherapy.

### Meta-analysis of the high level of GRP75 with prognosis

**Fig. 7:**
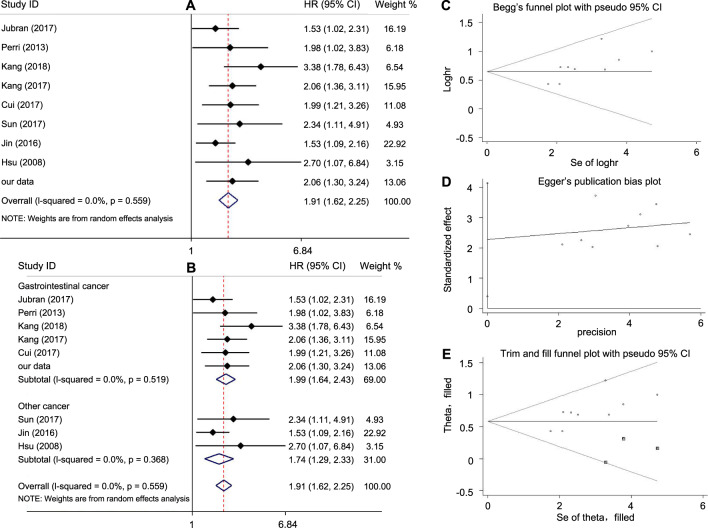
Meta-analysis of the high level of GRP75 with poor prognosis. **A** Frost blot of the association between overall survival and GRP75 expression. **B** Analysis based on cancer type, data were divided into gastrointestinal cancers and other cancers. **C** and **D** Begg’s and Egger’s publication bias plot of studies included in this analysis. **E** The trim and fill method to exclude publication bias.

Flow diagram of the literature search and selection and meta-analysis were shown in Supplementary Fig. S2. The random-effect model and fixed-effect model were used to calculate and analyze the HR value, both of them showed high levels of GRP75 are significantly associated with poor patient outcomes. The pooled HR was 1.91 (95% CI 1.62 to 2.25), with heterogeneity (*I*^2^ = 0.0%, *p* = 0.559) (Fig. 7A). Then we made a subgroup analysis, which showed that the expression level of GRP75 in gastrointestinal cancer (pooled HR was 1.99, 95% CI 1.64 to 2.43), has a significant relationship with poor survival prognosis. Certainly, similar results were also found in other tumors (pooled HR was 1.74, 95% CI 1.29 to 2.33) (Fig. 7B). Both Begg’s funnel plot and Egger’s test were used to assessing the possible publication bias of the included studies. In the analysis of the association between GRP75 and OS, the *p-*value of Begg’s test and Egger’s test were 0.076 and 0.024, respectively (Fig. 7C and D). However, Egger’s test has a higher sensitivity in evaluating publication bias. Thus, the trim and fill method was used to make our results more credible. As shown in Fig. 7E, the adjusted HR in the fixed-effect model was 1.785 (95% CI 1.530 to 2.081, *p* < 0.001), and in the random effect model was 1.792 (95% CI 1.515 to 2.120, *p* < 0.001), which was not significantly different from overall HR. In our analysis, a high level of GRP75 showed a poor prognosis including but not limited to gastrointestinal cancer, which was highly consistent with our research.

## Discussion

In this study, we used one of the first-line chemotherapeutic agents for patients with GC, platinum drugs. Classically, platinum drugs could covalently bind to guanine on the DNA chain and thus, block the replication and transcription of DNA^20^. However, because of drug resistance and undesirable side effects, identifying the novel therapeutic strategies to reverse resistance in GC patients was urgently essential. In addition, GC patients developed drug resistance after receiving several courses of cisplatin, and our results showed that SGC7901^CR^ cells exhibited significant resistance to cisplatin in comparison with SGC7901 cells.

GRP75 (mortalin/mot-2/HSPA9) played a key role in regulating the initiation and progression of human cancers^5–7^. More recently, it had become clear that GRP75 also had a critical role in chemotherapy resistance^8,9^. In addition to GRP75, other heat shock proteins played critical roles in cisplatin-resistance through PI3K/AKT/NF-κB and other pathways^21–24^. However, the molecular mechanism of GRP75 and cisplatin-resistance was rarely reported. Here, our study revealed that GRP75 promoted anti-oxidation/apoptosis abilities and altered metabolic reprogramming, leading to cisplatin-resistance in GC cells. We also found that GRP75 was upregulated in SGC7901^CR^ cells and in tissue samples from patients, and was correlated with drug resistance, pro-survival, growth, and poor outcomes. Meanwhile, multivariate analysis identified that GRP75 was an independent predictor for overall survival. Besides, meta-analysis indicated that a high level of GRP75 showed poor prognosis including but not limited to GC, which was highly consistent with our research. All of the above results validated that targeting GRP75 could be expected to become a new approach to reverse the cisplatin-resistance and improve the prognosis of GC patients.

Under physiological conditions, cells were inevitably exposed to ROS from external factors and intracellular aerobic metabolism. As a double-edged sword, appropriate ROS were important signal molecules that regulated the normal function of cells, while excessive ROS led to apoptosis. Therefore, a precise antioxidant regulation system existed in the body to maintain redox balance and apoptotic procedures^25,26^. However, unlike physiological conditions, ROS levels of tumors were generally significantly higher than normal controls of the same tissue origin, which determined the existence of a special antioxidant system in tumor cells, especially for drug-resistant tumor cells^27–29^. Our results confirmed that SGC7901^CR^ cells exhibited a relatively higher level of ROS in comparison with SGC7901 cells and that, GRP75 elevated the capacities of anti-oxidation/apoptosis.

Tumor cells had a long history of metabolic abnormalities. As early as the 1930s, Otto Warburg discovered that tumor cells prefer glycolysis. Even under oxygen-sufficient conditions, tumor cells still maintained high rates of glycolysis for ATP generation. This abnormal metabolic pattern was termed the “Warburg effect”^30,31^. The Warburg effect-led metabolic changes had recently been referred to as metabolic reprogramming, and studies of these changes provided a deeper understanding of GC cell metabolism. It had been demonstrated that GC cells and normal cells exhibited metabolic differences not only in glucose metabolism but also in the metabolism of lipids and amino acids^32–35^. In this research, we found that changes in glucose metabolism were one of the core links maintaining cell growth, eventually leading to GC cisplatin-resistance.

As we mentioned above, classical oncogenes and signaling pathways like PI3K/AKT, HIF-1α, and c-myc were involved directly or indirectly in metabolic reprogramming. Activation of the PI3K/AKT pathway in tumor cells enhanced lots of the metabolic activities. First, it permitted cells to the uptake of glucose, amino acids, and other nutrients. Second, AKT increased glycolysis and lactate production via its effects on gene expression and enzyme activity and was sufficient to induce a Warburg effect in cancer cells. Third, activation of this pathway enhanced the biosynthesis of macromolecules and stimulated the expression of lipogenic genes and lipid synthesis^17^. Furthermore, GRP75-activated AKT and extracellular signal-regulated protein kinases 1 and 2 inhibited Bax conformational changes and apoptosis^18^. Tumor cells adapted to hypoxia involving metabolic reprogramming via upregulating HIF-1α target genes to stimulate glucose uptake, glycolysis, production and secretion of lactic acid, glycogen storage, glutamine catabolism^36^, and promote the accumulation of triglycerides in lipid droplets^37^. More recently, it had become clear that GRP75 could bind specifically to HIF-1α and target to the outer mitochondrial membrane, associate with VDAC1 and HK2, which prevented apoptosis^9^. C-myc could mediate metabolic reprogramming in a number of ways, and c-myc-mediated metabolic reprogramming was largely achieved by affecting mitochondria. On the one hand, c-myc also promoted glycolysis by directly regulating the expression of glycolytic related enzymes, including LDHA, HK2, and PDK1^19,38^. On the other hand, c-myc activated enzymes involved in glutamine metabolism via transcription and promoted the utilization of glutamine by mitochondria in tumor cells. This effect is called “glutaminolysis”^39^. Moreover, GRP75-overexpression reduced Cyclin-B1 and upregulates Cyclin-D1 and c-myc to promote ovarian cancer cell growth^40^. Based on our present results, we provided a new understanding of GC cisplatin-resistance through GRP75 as a potentially important link in the regulation of tumor metabolic reprogramming networks.

## Conclusions

**Fig. 8:**
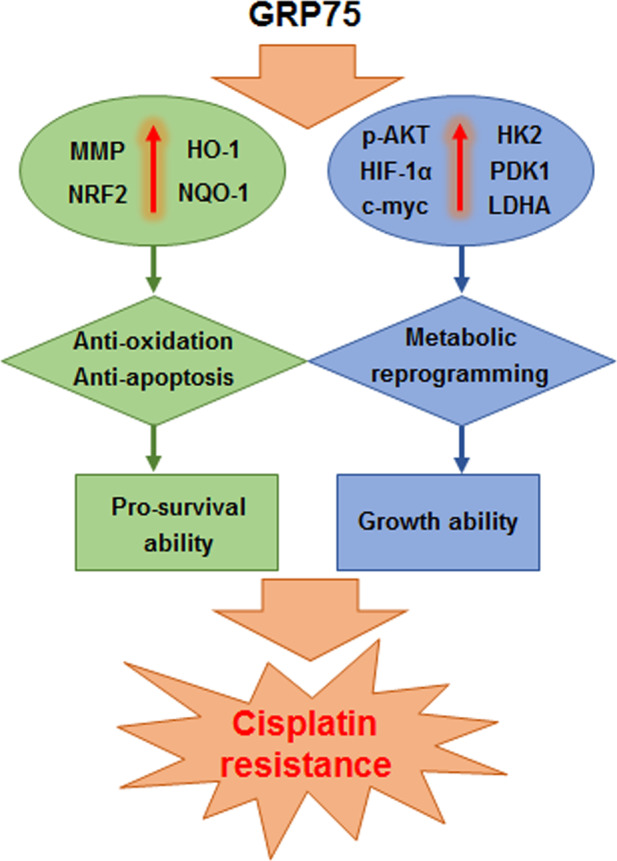
The possible mechanisms of GRP75 promoting the survival/growth of GC cells. A hypothetic scheme showed that overexpression of GRP75 could promote survival/growth in GC cells by regulating anti-oxidation/apoptosis and metabolic reprogramming networks, thereby inducing cisplatin-resistance.

In conclusion, we demonstrated that overexpression of GRP75 could promote survival/growth in GC cells by regulating anti-oxidation/apoptosis and metabolic reprogramming networks, thereby inducing cisplatin-resistance (Fig. 8). We also showed that GRP75 was upregulated in human GC and related to poor prognosis. Further, GRP75 could be a promising therapeutic target for the cisplatin-resistance of GC patients and a biomarker for predicting overall survival.

## Materials and methods

### Cells, reagents, and culture conditions

GC cell lines, SGC7901 cells (mutant-type of p53), and cisplatin-resistance cells (SGC7901^CR^) were obtained from and STR identified by KeyGENE Bio Co. Ltd (Nanjing, China). Cisplatin (Pt(NH_3_)_2_Cl_2_, >99.0% purity), were purchased from Sigma-Aldrich (Shanghai, China). Cells were cultured in RPMI-1640 medium (Gibco, Grand Island, NY, USA), supplemented with 10% fetal bovine serum (FBS), 100 U/ml penicillin, 100 μg/ml streptomycin (Gibco), and incubated in 5% CO_2_ at 37 °C. For maintenance of the cisplatin-resistance phenotype, SGC7901^CR^ cells were incubated in a medium containing cisplatin (5 μM).

### Xenografts and treatments

This study was approved by Nanjing Medical University Institutional Animal Care and Use Committee, and animals were treated humanely and with regard to the alleviation of suffering. The BALB/c nude mice were obtained from SLRC Laboratory Animal Center (Shanghai, China) and conventionally kept as we described previously^41^. For the xenograft study, 2 × 10^6^ SGC7901^CR^ cells in 100 μl matrigel were injected subcutaneously into the flanks of the mice for 5 weeks. To determine the effects of GRP75 on the cisplatin-resistance of GC, we performed the intratumoral and intraperitoneal injection assay. Briefly, mice were randomly divided into 4 groups (5 mice per group): (1) MOCK, (2) GRP75 KD, (3) MOCK + cisplatin, (4) GRP75 KD + cisplatin. 100 μl of siRNA (si-Con or si-GRP75, 100 nM) were intratumoral injections every 3 days. The groups subjected to cisplatin (5 mg/kg) therapy were intraperitoneal injection 3 times per week and the other groups were perfused with an equal volume of saline. Tumors were measured every week and their volumes were calculated using the formula: *V* = ½ (width^2^ × length). After 5 weeks, the mice were sacrificed, and tumor tissues were removed for further investigation.

### Patients and tissue specimens

This study was approved by the Medical Ethics Committee of the Second Affiliated Hospital, Nanjing Medical University, and the Affiliated Changzhou No. 2 Hospital of Nanjing Medical University and the participants’ written informed consents were obtained from each patient. The clinic-pathologic data were listed in Supplementary Table S1. The tissue microarray was constructed by Zhuoli Biotechnology Co. Ltd (Shanghai, China) as we described previously^41^.

### Cell transfection

For transfection, scrambled and pcDNA-3.1-GRP75-Flag were synthesized by Generay Biotech (Shanghai, China); while siRNAs were listed in Supplementary Table S2. Cells were transiently transfected via lipofectamine 3000 reagent (Invitrogen, Carlsbad, USA), according to the manufacturer’s protocol. Briefly, cells were plated onto 6-well plates at a density of 1 × 10^5^ cells in RPMI 1640 medium containing 10% FBS without antibiotics. After incubation for 24 h, the cells were transiently transfected with 5 ng/ml scrambled or GRP75-Flag, or 20 nM si-Con or si-GRP75 for 12 h. After transfection, the cells were cultured in a fresh medium supplemented with 10% FBS for another 24 h before being used for other experiments.

### Quantitative real-time polymerase chain reaction (qPCR)

The primers used were listed in Supplementary Table S3. The isolation of total RNA, the transcription of RNA to cDNA, and the performance of qRT-PCR with Applied Biosystems 7300HT machine were all according to our previous study^42^. The β-actin was amplified to ensure cDNA integrity and to normalize expression. Fold changes in expression of each gene were calculated by a comparative threshold cycle (Ct) method using the formula 2^-(ΔΔCt)^.

### Western blot

The antibodies used were listed in Supplementary Table S4. Extraction of total/nuclear proteins, measurement of their concentrations with BCA kit (Beyotime Co. Ltd., Nanjing), and SDS-PAGE followed by transferring the protein to PVDF membranes were all according to our previous study^43^.

### Cell viabilities and calculation of the 50% inhibitory concentrations (IC_50_)

Cell viabilities were determined by using a Cell Counting Kit-8 (CCK-8) (Beyotime Co. Ltd., Nanjing). The IC_50_s were calculated via a graph-pad 8.0 software (CA, USA). The determination of inhibition ratio, the selection of calculation mode, the generation of the sigmoidal curve, and the acquisition of IC_50_ value were all based on our previous description^29^.

### Bioinformatics analysis

The microarray raw data of GSE122130 and GSE14209 were downloaded from the Gene Expression Omnibus (GEO, https://www.ncbi.nlm.nih.gov/geo/). These data were arranged by using R language and were normalized by the “affy” package. The limma package was used to identify the differentially expressed genes (DEGs) between cisplatin-resistant and cisplatin-sensitive cell lines/tissues. The DEGs in GSE14209 were screened out according to *p*-value < 0.05 and |logFC|>0.26, and in GSE122130 were screened out according to |logFC|>1. The Database for Annotation, Visualization, and Integrated Discovery (DAVID; http://david.ncifcrf.gov) (version 6.8) was an online biological information database that integrates biological data and analysis tools^44^. Kyoto Encyclopedia of Genes and Genomes (KEGG) pathway and Gene Ontology (GO) terms were conducted to reveal functional and characteristic biological attributes of the DEGs based on DAVID online database^45,46^. *p*-value < 0.05 was set as the cut-off criterion for the significant enrichment. NetworkAnalyst (http://www.networkanalyst.ca), and R programming languages-based online tool, was used to analyze the protein-protein interaction (PPI) according to the KEGG database^47^. PPI network of 60 proteins which significantly interacted with GRP75 were constructed using the STRING database^48^ (version 11.0), and interaction with a combined score >0.4 was considered statistically significant. Cytoscape software (version 3.6.1) was used to visualize it^49^.

### Mitochondrial membrane potential (MMP) measurement

MMP of GC cells was measured by using fluorescent probe JC-1 (Beyotime Co. Ltd., Nanjing). The cells treated with cisplatin for 24 h were rinsed with PBS and incubated with 1 ml JC-1 staining working fluid at 37 °C for 20 min. Afterward, the cells were rinsed with JC-1 staining buffer twice. Fluorescent intensity of the JC-1 monomers and aggregates was detected under different conditions (Ex (*λ*) 490 nm, Em (*λ*) 530 nm for monomers; Ex (*λ*) 525 nm, Em (*λ*) 590 nm for aggregates) on a multi-well plate reader (Bio-Rad, USA).

### Intracellular reactive oxygen species (ROS) determination

The ROS Assay Kit was purchased from Beyotime Co. Ltd. As we described previously^29^, treated cells were incubated with DCFH-DA and the fluorescent signal was observed via a fluorescence microscope (Olympus, Tokyo, Japan), the DCFH fluorescence intensity was measured via a multi-well plate reader at Ex (*λ*) 488 nm and Em (*λ*) 525 nm (Bio-Rad, USA).

### Apoptosis assay

Cells were cultured in 6-well plates following treatment with cisplatin at 37 °C for 48 h. Cells were collected, washed twice with cold PBS, and re-suspended in 100 µl binding buffer containing 5 µl fluorescein isothiocyanate (FITC)-Annexin V and 5 µl PI using a FITC-Annexin V Apoptosis Detection kit (BD Biosciences, USA). The samples were assessed using a FACS Calibur flow cytometer (BD Cell Quest Pro, BD, Biosciences, USA).

### Analysis of caspase-3 activity

Cells were cultured and treated with cisplatin at 37 °C for 24 h. The activity of Caspase-3 was measured according to the specifications of the caspase-3 Activity Assay Kit (Beyotime Co. Ltd., Nanjing). Briefly, the detection samples were acquired by cell lysis and centrifugation at 4 °C. This assay was based on the principle that Ac-DEVD-pNA (acetyl-Asp-Glu-Val-Asp p-nitroanilide) is catalyzed by caspase-3 and then produces pNA (p-nitroaniline), which gives a yellow color. The caspase-3 activity was detected via a multi-well plate reader at 405 nm and was normalized and calculated as the percentage of the control group.

### Glucose uptake assay

Evaluation of glucose uptake ability in GC cells used the fluorescent glucose 2-NBDG (Thermo Fisher Scientific). GC cells cultured in 96-well plates without glucose or carbon sources following treatment with cisplatin for 6 h, respectively. The cells were gently rinsed with HBSS and incubated with 100 μM 2-NBDG at 37 °C for 30 min and then rewashed with HBSS. Fluorescent intensity was detected on a microplate reader (Ex (*λ*) 465 nm; Em (*λ*) 540 nm)^50^.

### Cell growth assay

For the determination of growth kinetics, 1 × 10^5^ cells were seeded in six-well plates, and cultured for 24 h with or without cisplatin. Cells were then collected and counted in triplicate using a hemocytometer under a microscope.

### Immunohistochemistry (IHC)

As we described previously^41^, sections mounted on silanized slides were dewaxed in xylene; dehydrated in ethanol; boiled in 0.01 M citrate buffer (pH 6.0) for 20 min in a microwave oven, and then incubated with 3% hydrogen peroxide for 5 min. After washing with PBS, sections were incubated in 10% normal bovine serum albumin for 5 min, followed by incubation with primary antibody at 4 °C overnight. The slides were then incubated with a horseradish peroxidase-conjugated secondary antibody at room temperature for another 30 min. Samples were then visualized using diaminobenzadine, dehydrated, cleared, mounted, and photographed under a panoramic-scan digital slice scanning system (3DHISTECH Co. Ltd., Budapest, Hungary). The graphs were analyzed using Image-Pro-Plus 6.0 software. The quantitation of immunostaining was performed by two independent researchers who were blinded to the patient. The scores of GRP75 immunohistochemistry (IHC)-intensity were presented as 0 point (none), 1 point (low), 2 points (medium), and 3 points (high). According to the GRP75 IHC-intensity, patients were divided into two groups. When the scores were ≥2, they were defined as the high group.

### Statistical analysis

Data were presented as the mean ± SD. The statistical significance of results was calculated using student’s *t*-test, two-way analysis of variance followed by Sidak’s multiple comparisons test via a graph-pad 8.0 software. Overall survival analysis was performed using the Kaplan–Meier method and log-rank test. Clinicopathological features were analyzed by a *χ*2 test. A Cox proportional hazards regression model was used to identify independent prognostic factors associated with overall survival. The *p-*value < 0.05 was defined as statistically significant.

## Supplementary information

Supplementary Figure S1

Supplementary Figure S2

Supplementary Figure Legends

Supplementary Tables
