# Supplementary figures and images for: Mortalin/glucose-regulated protein 75 promotes the cisplatin-resistance of gastric cancer via regulating anti-oxidation/apoptosis and metabolic reprogramming
Source: Cell Death Discov. 2021 Jun 11;7:140. doi: 10.1038/s41420-021-00517-w (PMC8196146; doi:10.1038/s41420-021-00517-w)

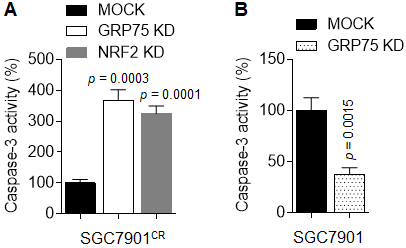

Supplement: Supplementary file 1 — Supplementary Figure S1 [file 41420_2021_517_MOESM1_ESM.tif]

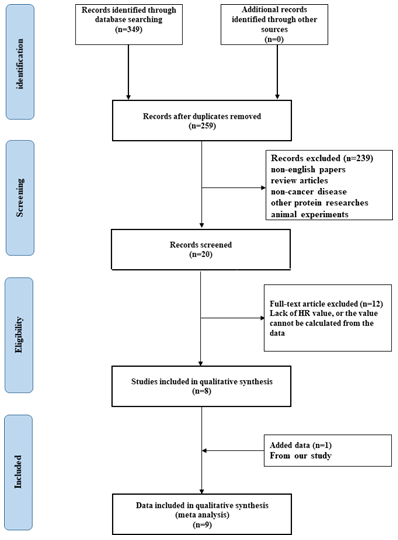

Supplement: Supplementary file 2 — Supplementary Figure S2 [file 41420_2021_517_MOESM2_ESM.tif]
