# Supplementary material for: Mortalin/glucose-regulated protein 75 promotes the cisplatin-resistance of gastric cancer via regulating anti-oxidation/apoptosis and metabolic reprogramming
Source: Cell Death Discov. 2021 Jun 11;7:140. doi: 10.1038/s41420-021-00517-w (PMC8196146; doi:10.1038/s41420-021-00517-w)
Supplement: Supplementary file 3 — Supplementary Figure Legends [file 41420_2021_517_MOESM3_ESM.docx]

**Supplementary figure legends**

**Figure S1. Effects of GRP75 on anti-apoptosis.**

**(A and B)** SGC7901^CR^ cells were transfected by scrambled or GRP75 siRNA, while SGC7901 cells were transfected by scrambled or GRP75 plasmids. After then, they were treated by 5 or 2.5 μM of cisplatin for 24 h. Caspase activity of GC cells were measured and calculated as percentage of control group.

**Figure S2. Flow diagram of the literature search and selection and meta-analysis.**

The following online databases were used for the data collection, PubMed, Web of Science and Cochrane library, Key search words used were: (1) prognosis OR prognostic OR survival. (2) cancer OR tumor OR tumour OR carcinoma OR neoplasm.(3) mot-2 OR HSPA9B OR GRP75 OR heat shock 70kDa protein 9B OR mortalin OR mortalin-2 OR heat shock 70kDa protein 9. The inclusion criteria were: (1) the cancers were diagnosed by the histological examination or any other accepted standard; (2) GRP75 was studied in human cancers;(3) the expression of GRP75 and the clinical outcome of patients were included in the research;(4) reports with survival outcome and the data analysis HR with 95% CI. And the exclusion criteria were: (1) non-neoplastic disease (2) repeat publications; (3) articles focused on other proteins; (4) case reports, reviews, letters and animal trails;(5) non-English papers;(6) lack of HR or insufficient data for estimating HR and 95% CIs. We started with 349 papers by using the above search strategies, at last 8 articles were included [^1-8^](#_ENREF_1). With our data, a total of 9 data were concluded in our analysis. Then, data was analyzed by using the STATA version 14.0 MP software. The heterogeneity between studies was performed on the basis of Q-test. If *p* value > 0.1 and I2 < 50%, we reputed no heterogeneity. HR > 1 indicates poor prognosis in patients. Publication bias was evaluated by the Begg’s test and Egger’s test. If the publication bias was exist, the trim and fill method was used to adjust the results.

**Supplementary references**

1 Fu, Y. & Lee, A. S. Glucose regulated proteins in cancer progression, drug resistance and immunotherapy. *Cancer biology & therapy* **5**, 741-744 (2006).

2 Gao, R., Singh, R., Kaul, Z., Kaul, S. C. & Wadhwa, R. Targeting of DNA Damage Signaling Pathway Induced Senescence and Reduced Migration of Cancer cells. *The journals of gerontology. Series A, Biological sciences and medical sciences* **70**, 701-713 (2015).

3 Kang, Q., Zou, H., Yang, X., Cai, J. B., Liu, L. X., Xie, N. et al. Characterization and prognostic significance of mortalin, Bcl-2 and Bax in intrahepatic cholangiocarcinoma. *Oncology letters* **15**, 2161-2168 (2018).

4 Kang, Q., Cai, J. B., Dong, R. Z., Liu, L. X., Zhang, C., Zhang, P. F. et al. Mortalin promotes cell proliferation and epithelial mesenchymal transition of intrahepatic cholangiocarcinoma cells in vitro. *Journal of clinical pathology* **70**, 677-683 (2017).

5 Sun, J., Che, S. L., Piao, J. J., Xu, M., Chen, L. Y. & Lin, Z. H. Mortalin overexpression predicts poor prognosis in early stage of non-small cell lung cancer. *Tumour biology : the journal of the International Society for Oncodevelopmental Biology and Medicine* **39**, 1010428317695918 (2017).

6 Cui, X., Li, Z., Piao, J., Li, J., Li, L., Lin, Z. et al. Mortalin expression in pancreatic cancer and its clinical and prognostic significance. *Human pathology* **64**, 171-178 (2017).

7 Jin, H., Ji, M., Chen, L., Liu, Q., Che, S., Xu, M. et al. The clinicopathological significance of Mortalin overexpression in invasive ductal carcinoma of breast. *Journal of experimental & clinical cancer research : CR* **35**, 42 (2016).

8 Hsu, W. M., Lee, H., Juan, H. F., Shih, Y. Y., Wang, B. J., Pan, C. Y. et al. Identification of GRP75 as an independent favorable prognostic marker of neuroblastoma by a proteomics analysis. *Clinical cancer research : an official journal of the American Association for Cancer Research* **14**, 6237-6245 (2008).
