# Supplementary material for: Mortalin/glucose-regulated protein 75 promotes the cisplatin-resistance of gastric cancer via regulating anti-oxidation/apoptosis and metabolic reprogramming
Source: Cell Death Discov. 2021 Jun 11;7:140. doi: 10.1038/s41420-021-00517-w (PMC8196146; doi:10.1038/s41420-021-00517-w)
Supplement: Supplementary file 4 — Supplementary Tables [file 41420_2021_517_MOESM4_ESM.docx]

**Supplementary TABLES**

**Table S1. GRP75 levels and clinicopathological factors in gastric cancer tissue specimens (n=116).** Note: All patients were received platinum-based chemotherapy.

| Factors | GRP75 expression | | *p* value |
| --- | --- | --- | --- |
|  | Low (n=58) | High (n=58) |  |
| Age  ≤60  >60 | 31 (53.4) 27 (44.6)  27 (44.6) 31 (53.4) | | 0.578 |
| Gender  Male  Female | 43 (74.1) 44 (75.9)  15 (25.9) 14 (24.1) | | 1.000 |
| Tumor size  ≤5 cm  >5cm | 38 (65.5) 19 (32.8)  20 (34.5) 39 (67.2) | | 0.0008 |
| Differentiation  Well  Poor | 32 (55.2) 19 (32.8)  26 (44.8) 39 (61.2) | | 0.0248 |
| Vascular invasion  Negative  Positive | 48 (82.8) 41 (70.7)  10 (17.2) 17 (29.3) | | 0.187 |
| pTNM stage  I/II  III | 30 (44.6) 11 (18.9)  28 (53.4) 47 (81.1) | | 0.0004 |

**Table S2. siRNAs used in this study.**

| Names | Web Link | Source | Used |
| --- | --- | --- | --- |
| GRP75  siRNA | https://datasheets.scbt.com/sc-35520.pdf | Santa Cruz Biotechnology | 20 nM |
| NC  siRNA | <http://datasheets.scbt.com/sc-37007.pdf> | Santa Cruz Biotechnology | 20 nM |
| NRF2  siRNA | https://datasheets.scbt.com/sc-37030.pdf | Santa Cruz Biotechnology | 20 nM |
| AKT  siRNA | https://datasheets.scbt.com/sc-43609.pdf | Santa Cruz Biotechnology | 20 nM |

**Table S3. Primers used in this study.**

| Names | Primers |
| --- | --- |
| GRP75  β-Actin  PDK1  HK2  LDHA  HO-1  NQO-1 | F: 5’-GCTGTCAATCCTGATGAGGCTGTG-3’  R: 5’-CTGGCTCTTCTTGGTTGGAATAGTGG-3’  F: 5’-TCAGGTCATCACTATCGGCAAT-3’  R: 5’-AAAGAAAGGGTGTAAAACGCA-3’  F: 5’-GATGTGAATGGGCAGTTAGT-3’  R: 5’-AGGAATAGTGGGTTAGGTGAG-3’  F: 5’-CAAAGTGACAGTGGGTGTGG-3’  R: 5’-GCCAGGTCCTTCACTGTCTC-3’  F: 5’-TGGAGTGGAATGAATGTTG-3’  R: 5’-GATGTGTAGCCTTTGAGTTTG-3’  F: 5’-AGCAGGACATGGCCTTCT-3’  R: 5’-TCTGTCAG CATCACCTGCAG-3’  F: 5’-CCATTCTGAAAGGCTGGTTTG-3’  R: 5’-CTAGCTTTGATCTGGTTGTC-3’ |

**Table S4. Antibodies used in this study.**

| Names | Web Link | Source | Used |
| --- | --- | --- | --- |
| GRP75 | https://www.cst-c.com/products/primary-antibodies/grp75-d13h4-xp-rabbit-mab/3593?site-search-type=Products | Cell Signaling Technology | 1: 1000 |
| HIF-1α | https://www.cst-c.com/products/primary-antibodies/hif-1a-d1s7w-xp-rabbit-mab/36169?site-search-type=Products | Cell Signaling Technology | 1: 1000 |
| p-AKT | https://www.cst-c.com.cn/products/primary-antibodies/phospho-akt-ser473-d9e-xp-rabbit-mab/4060?site-search-type=Products | Cell Signaling Technology | 1: 1000 |
| c-myc | https://www.cst-c.com.cn/products/primary-antibodies/c-myc-e5q6w-rabbit-mab/18583?site-search-type=Products | Cell Signaling Technology | 1: 1000 |
| NRF2 | https://www.cst-c.com.cn/products/primary-antibodies/nrf2-d1z9c-xp-rabbit-mab/12721 | Cell Signaling Technology | 1: 1000 |
| β-Actin | https://www.cst-c.com/products/primary-antibodies/b-actin-13e5-rabbit-mab/4970?site-search-type=Products | Cell Signaling Technology | 1:1000 |
